# Screening of 10 DFNB Loci Causing Autosomal Recessive Non-Syndromic Hearing Loss in Two Iranian Populations Negative for *GJB2* Mutations

**Published:** 2019-09

**Authors:** Mahbobeh KOOHIYAN, Somayeh REIISI, Fatemeh AZADEGAN-DEHKORDI, Mansoor SALEHI, Hamidreza ABTAHI, Morteza HASHEMZADEH-CHALESHTORI, Mohammad Reza NOORI-DALOII, Mohammad Amin TABATABAIEFAR

**Affiliations:** 1. Department of Genetics and Molecular Biology, School of Medicine, Isfahan University of Medical Sciences, Isfahan, Iran; 2. Department of Genetics, Faculty of Basic Sciences, University of Shahrekord, Shahrekord, Iran; 3. Cellular and Molecular Research Center, Basic Health Sciences Institute, Shahrekord University of Medical Sciences, Shahrekord, Iran; 4. Department of Otolaryngology, Al-Zahra Hospital, Isfahan University of Medical Sciences, Isfahan, Iran; 5. Department of Medical Genetics, School of Medicine, Tehran University of Medical Sciences, Tehran, Iran; 6. Pediatric Inherited Diseases Research Center, Research Institute for Primordial Prevention of Noncommunicable Diseases, Isfahan University of Medical Sciences, Isfahan, Iran

**Keywords:** Autosomal recessive non-syndromic hearing loss (ARNSHL), DFNB loci, Homozygosity mapping, Iran

## Abstract

**Background:**

Autosomal recessive non-syndromic hearing loss (ARNSHL), one of the global public health concerns, is marked by a high degree of genetic heterogeneity. The role of *GJB2,* as the most common cause of ARNSHL, is only <20% in the Iranian population. Here, we aimed to determine the relative contribution of several apparently most common loci in a cohort of ARNSHL Iranian families that were negative for the *GJB2* mutations.

**Methods:**

Totally, 80 Iranian ARNSHL families with 3 or more affected individuals from Isfahan and Hamedan provinces, Iran were enrolled in 2017. After excluding mutations in the *GJB2* gene via Sanger sequencing, 60 negative samples (30 families from each province) were analyzed using homozygosity mapping for 10 ARNSHL loci.

**Results:**

Fourteen families were found to be linked to five different known loci, including DFNB4 (5 families), DFNB2 (3 families), DFNB7/11 (1 family), DFNB9 (2 families) and DFNB3 (3 families).

**Conclusion:**

Despite the high heterogeneity of ARNSHL, the genetic causes were determined in 23.5% of the studied families using homozygosity mapping. This data gives an overview of the ARNSHL etiology in the center and west of Iran, used to establish a diagnostic gene panel including most common loci for hearing loss diagnostics.

## Introduction

Hearing loss (HL) is the most frequent form of sensory impairment in humans, with approximately 1–2 in 1000 infants being born with a serious HL (https://www.gov.uk/guidance/newborn-hearing-screening-programme-overview). Over 70% of prelingual HL cases can be classified as non-syndromic HL (NSHL), where the hearing deficit is the only sign (1). HL can be inherited as autosomal recessive, autosomal dominant, mitochondrial, X- and Y-linked. Autosomal recessive mode of inheritance (ARNSHL) makes up 80% of the NSHL cases. ARNSHL is highly heterogeneous, for which over 100 mapped loci are known. The autosomal recessive loci are called DFNB followed by a number corresponding to the order that the locus was first explained; DFNB1 to DFNB105 have been reported so far (http://hereditaryhearingloss.org). However, more than seventy different DFNB loci have been mapped for ARNSHL by homozygosity mapping, an effective method to locate genes causing ARNSHL in large consanguineous families (2).

The genetic etiology of ARNSHL in Iran has been studied by a number of independent studies with a special focus on the certain locus DFNB1 (*GJB2*), as the most common cause of HL all over the world (3). Variants in the *GJB2* (NM_004004.5) could explain the etiology of ARNSHL in 4% to 35% of patients from different origin, suggesting that GJB2 gene mutations account for a part of ARNSHL in Iran. (4, 5). On the other hand, Iran is a large country with many ethnic groups and high rate of the consanguineous marriages (38.6% on average) (6). Thus, mutations in certain loci may be increased within some ethnic groups (7). Therefore, population-specific studies are necessary to identify other common loci and to determine the etiology of ARNSHL based on ethnicity. Until now, little data is available on the contribution of some frequent loci among the Iranian ARNSHL; and have mostly focused on mixed populations from different provinces (8). Mutations in at least 40 genes have been explained to cause ARNSHL in Iran. However, the contribution of their mutations does not appear to occur at the same frequencies across different ethnic groups (9).

We applied the homozygosity mapping strategy to identify the relative contribution of 10 DFNB loci to ARNSHL in the studied cohort including 60 ARNSHL families, which were negative for *GJB2* mutations, from Isfahan and Hamedan provinces for the first time. This data could be applied to design a cheap and accurate panel for common genes causing ARNSHL in certain regions of Iran and would lead to affordable testing and improved management of HL (10).

## Materials and Methods

### Families and clinical evaluations

Totally, 60 unrelated consanguineous families with 3 or more hearing impaired patients and negative for *GJB2* mutations were recruited from Isfahan and Hamedan provinces in the center and west of Iran in 2017.

The Ethics Committee of the Isfahan University of Medical Sciences approved this project. All family members signed informed written consent prior to recruitment. They met the following criteria: 1) confirmation of HL by Pure tone audiometry (PTA) from 250–8000 Hz 2) the autosomal recessive inheritance deduced through pedigree analysis 3) existence of three or more affected members within the pedigree. A complete clinical investigation was performed to exclude environmental exposures and to determine the presence of syndromic findings in each family.

### DNA extraction

Genomic DNA was extracted using Prime Prep Genomic DNA Extraction kit from blood (GeNet Bio, Korea) according to the manufacturer’s instruction. Qualitative and quantitative assessment of genomic DNA was checked using 1.2% agarose gel and Nanospec cube biophotometer (Nanolytik®, Dusseldorf, Germany).

### SLINK analysis and selection of DFNB loci

Power of the pedigrees for linkage analysis was simulated by calculating SLINK, using FastSLink (ver.2.51) option of Easy linkage plus version 5.05 software to predict the potential LOD score in a given family (11). Based on the literature review of the most frequent loci, both globally and regionally, 10 loci were selected for screening. Screening sort tandem repeat (STR) markers were selected based on their physical distance found at NCBI UniSTS and NCBI Map Viewer (http://www.ncbi.nlm.nih.gov). Primers for STR markers of each locus were mostly obtained through the Probe database (https://www.ncbi.nlm.nih.gov/probe). STR markers of each locus and their primer sequences are listed in Table 1.

**Table 1:** The list of 10 DFNB loci screened in this study. The corresponding genes and characteristic of screening markers are shown

***locus (gene)***	***STR***	***Heterozygosity (%)***	***Size(bp)***	***Forward primer***	***Reverse primer***
DFNB7–11 (*TMC1*)	D9S1876	81	132–152	GATGTACCCAGAGAAGTCTCG	AGTGGTTACCATTTACCCAAG
	D9S1124	76	252–276	GGTGCCCACCATACACTACT	TCTAATCCTTCCTTCCCTCG
	D9S301	78	205–251	CATGATGGTGGTCTCTGG	GGTGGGGCTCAAAGAGTAG
	D9S1799	91	139–178	TTGCCAACTATTTTAGCCC	TGCAGTTTCAATCCACATC
DFNB3 *(MYO15A*)	D17S953	81	119–131	ACTATCCGCCCAATACA	AAGGGCTTGCTTTGAC
	D17S1843	70	177–187	TGCACAGGCCAATTCCTTAC	TGCCTAAACTGCTTTCAGGTGAG
	D17S620	50	103–151	CTCTTTGTGCTTGGCAGGGT	TACATTTAATGCAGGATGCC
	D17S2196	81	139–163	CCAACATCTAGAATTAATCAGAATC	ATATTTCAATATTGTAACCAGTCCC
DFNB2 (*MYO7A)*	D11S4179	72	200–256	GGATGTAAGAGTAACTGG CTCCG	GAAAATGTTCTGCCTGAGGG
	D11S4186	79	154–175	ATTCTCCCAATCTATCGCTC	GGGCAGTAATGATGATGTG
	D11S4079	75	217–265	CAGCAAGATCCTGTCTCAA	CTCCTTAAAGTGGGGGAGTT
	D11S911	85	159–203	CTTCTCATGCTTGACCATTT	CTTCTGAACAATTGCCACAT
DFNB4 *(SLC26A4)*	D7S2420	81	240–290	CCTGTATGGAGGGCAAACTA	AAATAATGACTGAGGCTCAAAACA
	D7S496	63	129–141	AACAACAGTCAACCCACAAT	GCTATAACCTCATAANAAACCAAAA
	D7S2459	77	140–152	AAGAAGTGCATTGAGACTCC	CCGCCTTAGTAAAACCC
	D7S2456	78	238–252	CTGGAAATTGACCTGAAACCTT	ACAGGGGTCTCTCACACATATTA
DFNB9 (*OTOF)*	D2S365	85	164–204	ATGATTTGTGTACCTTATGTATGTT	TCAATGGAGGAATCCTACTT
	D2S2247	78	130–160	TCCATCTTTTGCGTGC	CCGTGCTCTATGCCAG
	D2S174	65	203–221	AGGCTGAATCCCACCTCC	TTAGAGCACACATGGTCACTCC
	D2S2223	63	182–200	CACTGCGCCTAGCCTC	GGCGATTTATGAATAATCCTGC
DFNB21 *(TECTA*)	D11S1774	60	206–226	CAAAAAGGCTTGGCGGTT	GGGCATTCCCATGCTCA
	D11S925	85	173–195	AGAACCAAGGTCGTAAGTCCTG	TTAGACCATTATGGGGGCAA
	D11S4089	75	199–213	TAATCAAAGGCTGTAGTGAATTGG	ATTCCTAGTTCCCTCATAAACACTG
	D11S4107	70	172–212	TCATTCTACAAGACTAGCATTACC	GCTTGATCATGGTGTATTATCTT
DFNB53 (*CO11A2l)*	D13S1236	70	108–132	GCACTTGGCCTGGGTAA	AAGGGGCTGGCTCTTCA
	D13S175	75	101–113	TATTGGATACTTGAATCTGCTG	TGCATCACCTCACATAGGTTA
DFNB59 *(PJVK)*	D2S2173	70	201–243	GGAGACAGAGAGTTTACATTTGAG	GCCACACTTTCCTGAATC
	D2S324	85	264–275	TTACCCACCGGGACAGT	CAGCAAATGCTTCTAGGTCA
	D11S1314	78	209–227	TTGCTACGCACTCCTCTACT	GTGAAGGCAGGAAATGTGAC
DFNB63 *(LRTOMT)*	D11S4162	75	263–269	GTTCTCCAGAGAGACAGCAC	GAGAGCAACACTATTGCCC
	D11S4140	72	189–199	TGCAACAAGGTTCCACACT	CTTATGGGTGAGGGCACAG
	D11S4184	60	263–277	CCCAGCCTTACATATTCC	GCTGATGAGCAGAGGTAG
DFNB24 *(RDX)*	11S1793	85	124–140	AGTCATGCATCCTCCCTGTA	ATCCTGAACACATTCCTCAA
	D11S1391	75	158–178	TGCATGCATACATACATACATACA	CATCCATCCCTCTGTCTCTG
	D11S2017	70	109–133	TTTGAATAGGAAATTAGATGGTAGG	TTTGAATAGGAAATTAGATGGTAGG
	D11S1893	58	206–258	TCCCTGGAACCTGGAT	TGATGTGGGCTTTTTCAA

### Genotyping STR markers and Linkage Analysis

PCR of STR markers was conducted according to the standard procedure. The touchdown program was used for markers amplification. Thermal cycling conditions for amplifying markers were in accordance with previous protocols (4). PCR conditions were as follows: 2 μl MgCl_2_ (4 mM), 2.5 μl Taq PCR buffer (10X), 0.12 μl of each of the primers (10 PM), 0.15 μl Taq DNA polymerase (5U/ul), 0.9 μl dNTP mix (10 mM) and 1.2 μl DNA (about 70 ng). The reaction was adjusted to the volume of 25 ul by ddH_2_O. Standard cycling was done in a thermocycler (ASTEC PC-818; ASTEC, Fukouka, Japan). STR markers were selected based on their physical distance found at NCBI UniSTS. The criteria for selecting these markers are as follows: greater heterogeneity values, and lying near or at the known loci. At least 4 STR markers were selected for linkage analysis. Table 1 summarizes the general characteristics of the markers used in the study. PCR products were loaded on 12% polyacrylamide gel, followed by silver nitrate staining. Two-point and multipoint parametric LOD scores were calculated using Superlink (ver. 1.6) and GeneHunter (ver. 2.91), respectively. Haplopainter version 029.5 software package was used for reconstruction of haplotypes (12).

## Results

### Families and clinical data

After excluding mutations in the *GJB2* gene, 60 Iranian families segregating (ARNSHL), from Isfahan and Hamedan provinces (30 families from each province) in the center and west of Iran, were screened for 10 ARNSHL loci. Totally, 634 individuals were studied 245 of whom were patients, with ages ranging from 6 months to 52 yr. For 45 families, PTA was consistent with profound HL (≥80 dB), 12 families showed severe HL (61–80 dB), whereas the 3 remaining families showed moderate HL (41–60 dB).

### SLINK calculation, genotyping and linkage analysis

**Fig. 1:**
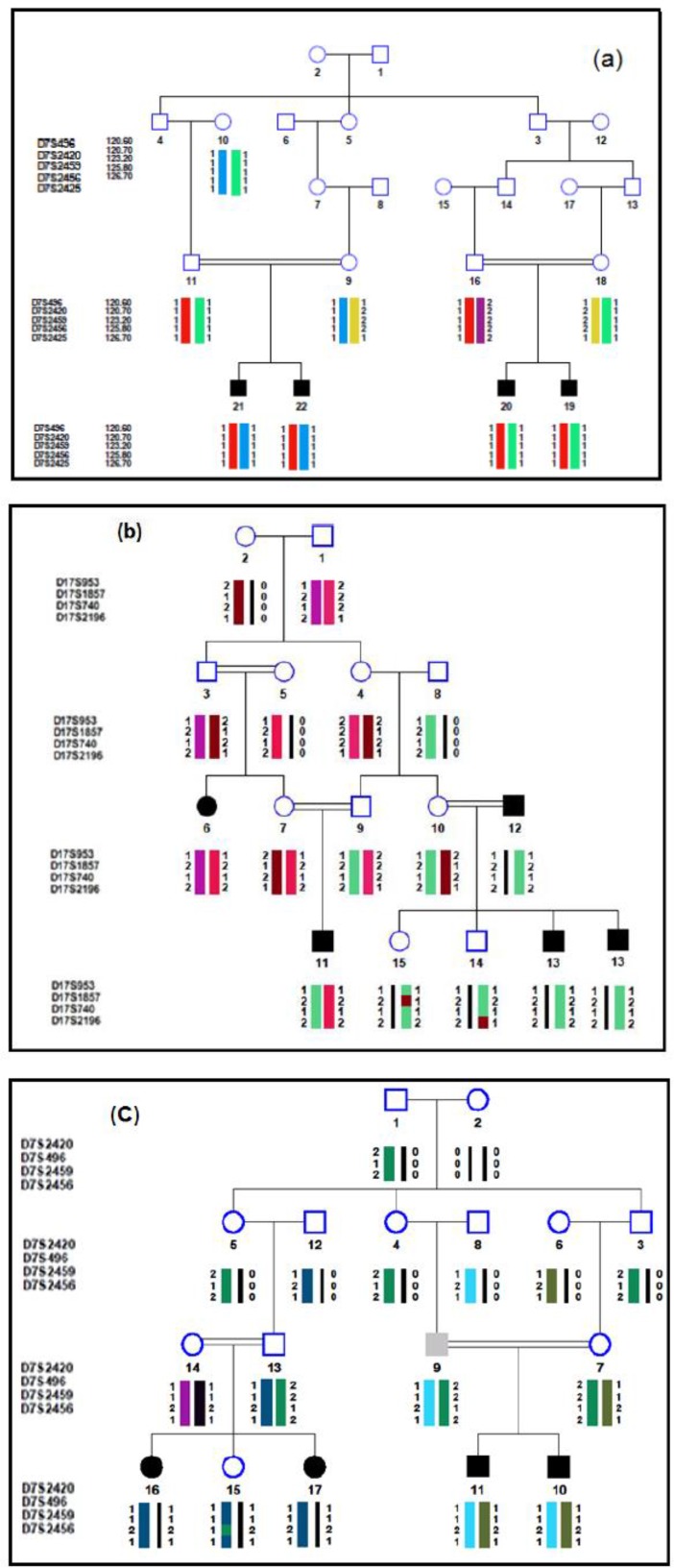
Pedigree and haplotypes of the 3 Iranian families with ARNSHL, negative for *GJB2* mutations, linked to 2 known loci. a) ISF-5 is linked to DFNB4: b) IR-3 is linked to DFNB3 c) IR-14 is linked to DFNB4. Hearing impaired patients show homozygosity for markers. The order of markers is based on the Marshfield map

Totally, 9 families were of SLINK values ≥3.2, 24 families had SLINK values of 2.5–3.2. The rest of the families presented values 1.8–2.5. Screening loci for homozygosity mapping in this study were composed of: DFNB2 (*MYO7A*), DFNB3 (*MYO15A*)*,* DFNB4 (*SLC26A4*), DFNB7/11(*TMC1*), DFNB9 (*OTOF*), DFNB21 (*TECTA*)*,* DFNB24 (*RDX*), DFNB59 (*PJVK*), DFNB63 *(LRTOMT)* and DFNB53 *(COL11A2).* The family members were individually genotyped for these markers. After genotyping of STR markers and linkage analysis, 14 out of the 60 families, negative for *GJB2* mutations, showed linkage to five different loci (Table 2). DFNB4 was the most frequent locus in the studied ARNSHL series in both provinces (36.9% of the etiology). Three out of 30 (10%) and 2 of 30 (6.6%) families were linked to DFNB4 in Isfahan and Hamedan provinces, respectively. DFNB2, DFNB3, and DFNB9 were ranked next after DFNB4. Table 3 shows the linked families and the maximum values for SLINK, two-point and multipoint LOD scores. One family (3.3%) from every single province was linked to DFNB9. One family from Hamedan Province was linked to DFNB7/11 (7.7% of the etiology). The haplotypes of 3 selected linked families are shown in Fig. 1(a–c).

**Table 2:** Genetic etiology of ARNSHL in 60 families negative for *GJB2* mutations from Isfahan and Hamedan provinces

***Number***	***Locus***	***Gene***	***Number of diagnoses***		***% of diagnoses***		***% of cohort***	
			***Isfahan***	***Hamedan***	***Isfahan***	***Hamedan***	***Isfahan***	***Hamedan***
1	DFNB2	*MYO7A*	1	2	7.2	15.4	3.3	6.6
2	DFNB3	*MYO15A*	1	2	7.2	15.4	3.3	6.6
3	DFNB4	*SLC26A4*	3	2	21.5	15.4	10	6.6
4	DFNB7-11	*TMC1*	0	1	0	7.7	0	3.3
5	DFNB9	*OTOF*	1	1	7.2	7.2	3.3	3.3
Total			6	8			19.9	26.4

**Table 3:** Maximum SLINK and LOD score (two-point and multi-point) values for the linked families

***Number***	***Family ID***	***SLINK value***	***Linked locus***	***Two-point LOD score***	***Multi-point LOD score***	***Severity HL***
	ISF-5	2.82	DFNB4	1.7	2.2	Severe-profound
2	ISF-15	2.9	DFNB4	2.6	2.8	Moderate-profound
3	ISF-6	3.8	DFNB4	3.1	3.2	Moderate-severe
4	IR-14	3.28	DFNB4	2.4	2.6	Profound
5	IR-9	1.8	DFNB4	2.2	2.3	Severe-profound
6	IR-13	2.53	DFNB2	1.9	2.3	Severe-profound
7	IR-30	2.8	DFNB2	2.3	2.6	Moderate-profound
8	ISF-17	1.9	DFNB2	2	2.2	Profound
9	IR-19	3.4	DFNB9	2.8	3.9	Severe-profound
10	ISF-14	2.8	DFNB9	1.9	2.3	Profound
11	IR-3	2.3	DFNB3	2.1	2.4	Profound
12	IR-27	3.1	DFNB3	2.9	3.1	Profound
13	ISF-23	2.4	DFNB3	2.1	2.3	Severe-profound
14	IR-7	2.1	DFNB7-11	2	2.2	Severe-profound

We did not find linkage to the other loci (DFNB21, DFNB24, DFNB59, DFNB63 and FNB53) among the studied families.

## Discussion

We determined the contribution of 10 DFNB loci to HL in 60 Iranian families affected with ARNSHL and negative for the *GJB2* mutations. We have obtained 20% and 26% involvement of *GJB2* mutations in ARNSHL in Hamedan and Isfahan provinces, respectively (unpublished data).

For the first time, we investigated the role of 10 other loci in the etiology of HL in *GJB2* negative families in the west and center of Iran. DFNB4 was found to be the mostly linked locus among the studied families, with 3 out of 30 (10%) and 2 of 30 (6.6%) showing linkage to it in Isfahan and Hamedan provinces, respectively.

Mutations in its corresponding gene (i.e. *SLC26A4*) are the second most common cause of ARNSHL, after *GJB2*, all over the world (13). About 5% of ARNSHL cases in South Asia have been related to *SLC26A4* mutations (14). Variants in this gene can cause both ARNSHL at the DFNB4 locus and Pendred Syndrome (PS), known as one of the most common forms of syndromic HL. PS is correlated to hypothyroidism (goiter) later in life. Present data show no sign of PS in the studied families. Several reports have revealed *SLC26A4* mutations in ARNSHL in Iranian populations. In a study, 12 families out of 80 (15%) Iranian families with 2 or more ARNSHL patients were linked to DFNB4 locus (15). In a recent study 12 out of 121 (9.9%) families were linked to DFNB4 (16). Thus, our result shows that DFNB4 contributes mainly to ARNSHL in the west and center of Iran and is ranked second after DFNB1, which is in agreement with previous studies reported from Iran (Table 4). The next most frequently linked locus in our cohort was DFNB2, which accounts for about 6.6% of HL etiology in Hamedan and 3.3% in Isfahan. The related gene *MYO7A*, encoding myosin VIIA, is an action-based molecular motor converting energy from ATP hydrolysis into mechanical force. It also interacts with actin to participate in the formation of the mechanotransduction complex, which is critical for detecting sound (17).

**Table 4:** The overview of DFNB loci frequencies obtained from in the studies on the Iranian ARNSHL patients

***Loci***	***Number of cases***	***Number of affected***	***Frequency (%)***	***References***
DFNB2	144	4	2.77	Babanejad et al.
	302	15	4.96	Heggen et al.
	60	3	5	This study
DFNB3	144	8	5.5	Babanejad et al.
	302	29	9.6	Heggen et al.
	60	3	5	This study
DFNB4	80	12	15	Kahrizi et al.
	302	37	12.25	Heggen et al.
	60	5	8.3	This study
DFNB7-11	144	4	2.7	Babanejad et al.
	54	1	2.2	Dahaghani et al.
	60	1	1.66	This study
DFNB9	144	1	0.69	Babanejad et al.
	38	1	2.38	Mahdieh et al.
	60	2	3.3	This study

DFNB2-linked families have been reported from Iran (18). 2.8% of DFNB2 involvement in ARNSHL etiology were reported in the studied Iranian cohort (19). Similarly, Sloan-Heggen et al. (20) using a custom targeted genomic enrichment method in a cohort of 302 *GJB2-*negative Iranian families, found the DFNB2 contribution to be 5%, which is in accordance with our results (Table 4).

Our data show that one out of 30 families (3.3%) from Isfahan province and two out of 30 families (6.6%) from Hamedan were linked to DFNB3. The DFNB3 locus was first identified in the village of Indonesia, Bengkala, with a frequency of 9.4% among the inbred population (21). Since then, many mutations have been reported from different countries such as India, Iran, Turkey, and Brazil (22, 23). In Pakistan, DFNB3 is the third locus for ARNSHL accounting for 5% of ARNSHL (24). In a previous study on 40 Iranian ARNSHL families from Qom and Markazi provinces, from center of Iran, 2 families were linked to DFNB3 (5.8%) (25). Therefore, the locus could be one common cause of ARNSHL both in the west and center of Iran.

In the present study, one family (3.3%) from every single province was linked to DFNB9 *(OTOF)*. Mutations in the *OTOF* gene (named as homology to Ferlin (Fer-1)) encoding otoferlin at DFNB9 results in ARNSHL which is sometimes associated with auditory neuropathy. Otoferlin is a member of the ferlin protein family; its role is in vesicle recycling and efficient and linear encoding of low-intensity stimulate the synapse between inner hair cells and auditory nerve fibers (26). *OTOF* mutations have been reported from many countries such as Pakistan, Spain, Italy, and Japan with different mutation spectrum (26, 27). The frequency of the *OTOF* gene mutations has been reported to be 2.7% in Iran (28). It was screened 37 Iranian ARNSHL families from 7 different provinces for 15 loci using linkage analysis and found 1 family (2.7%) to be linked to the DFNB9 locus. Similarly, in our present study, one family (3.3%) from each of the two provinces was linked to DFNB9 *(OTOF)*.

Our study involves 3 families with moderate to severe HL highlighted by a “U” shaped audiogram. DFNB21 and DFNB93 are related to this audio profile (18, 29). Interestingly, none of the 3 families was linked to DFNB21. Thus, the next step for these families involves investigating the DFNB93 contribution (30).

The contribution of DFNB7/11 in Hamedan Province was found to be 3.3% (1/30), in line with the prevalence figures of 4% in Pakistan, and 3.4% in Turkey. The related gene *TMC1* (Transmembrane channel-like gene I) is required for postnatal hair cell development. *TMC1* might be an ion channel or transporter which mediates K^+^ homeostasis in the inner ear (31). The *TMC1* gene was initially mapped to chromosome 9q13–q21 in two consanguineous Indian families with prelingual, severe-to-profound defining the DFNB7/11 locus (NM_138691.2). In the study on 144 *GJB2*-negative subjects, using linkage analysis and direct sequencing, 4 out of 144 families (2.7%) were linked to DFNB7-11(19). Moreover, Dehaghani et al. (32), using homozygosity mapping on 45 ARNSHL families, detected 1family (2.2%) to be linked to DFNB7/11. Therefore, the locus could be one common causes of ARNSHL in the west of Iran.

The next step of the study involves DNA sequencing of the corresponding genes of the DFNB loci in the linked families to identify the pathogenic mutations. Until now, 4 homozygous mutations have been identified in the related families, including 3 missense and 1 splice site mutations (7, 33). The identified mutations and their characteristics are listed in Table 5. The large size of some of the related genes hinders detection of their related mutations (34). However, the remaining families have been considered for sequencing of the related genes.

**Table 5:** The mutations detected in the linked families in this study

***Number***	***Family ID***	***Gene***	***Type of mutation***	***Nucleotide change***	***Amino acid change***
1	IR-14	*SLC26A4*	missense	c.416 G>T	p.Gly139Val
2	ISF-5	*SLC26A4*	Splice site	c.919-2 A>G	-
3	IR-27	*MYO15A*	missense	c.6442 T>A	p.Trp2148Arg
4	IR-13	*MYO7A*	missense	c.6487 G>A	p.Gly2163Ser

In the present study, despite the high heterogeneity of ARNSHL, we could detect the genetic etiology in 6 out of 30 (20%) and 8 out of 30 (26.6%) ARNSHL families, negative for *GJB2* mutations, from Isfahan and Hamedan provinces, respectively. Thus, nearly 23.5% of ARNSHL families negative for *GJB2* mutations are linked to 5 loci including DFNB4, DFNB2, DFNB3, DFNB9 and DFNB7/11. The remaining genes and loci could be either rare or yet to be identified (2, 28). The study is in progress by subjecting some of the remaining families to the next-generation sequencing, which is a rapid and cost-effect method.

## Conclusion

Using homozygosity mapping, we detected 23.5% of the genetic etiology of *GJB2-* mutation negative ARNSHL in Isfahan and Hamedan provinces. This finding is interesting in view of the extreme genetic heterogeneity of ARNSHL (with over 50 genes discovered so far). Investigation of a limited number of genes could lead to an acceptable level of diagnostic yield.

## Ethical considerations

Ethical issues (Including plagiarism, informed consent, misconduct, data fabrication and/or falsification, double publication and/or submission, redundancy, etc.) have been completely observed by the authors.
